# Failure Mechanisms of Cu–Cu Bumps under Thermal Cycling

**DOI:** 10.3390/ma14195522

**Published:** 2021-09-24

**Authors:** Kai-Cheng Shie, Po-Ning Hsu, Yu-Jin Li, Dinh-Phuc Tran, Chih Chen

**Affiliations:** 1 Department of Materials Science and Engineering, National Yang Ming Chiao Tung University, Hsinchu 30010, Taiwan; 911666alex@gmail.com (K.-C.S.); seraph8938@gmail.com (P.-N.H.); r777719982003@yahoo.com.tw (Y.-J.L.); trandinhphuc1508@gmail.com (D.-P.T.); 2 Department of Materials Science and Engineering, National Chiao Tung University, Hsinchu 30010, Taiwan

**Keywords:** Cu-to-Cu direct bonding, instant bonding, thermal cycling test

## Abstract

The failure mechanisms of Cu–Cu bumps under thermal cycling test (TCT) were investigated. The resistance change of Cu–Cu bumps in chip corners was less than 20% after 1000 thermal cycles. Many cracks were found at the center of the bonding interface, assumed to be a result of weak grain boundaries. Finite element analysis (FEA) was performed to simulate the stress distribution under thermal cycling. The results show that the maximum stress was located close to the Cu redistribution lines (RDLs). With the TiW adhesion layer between the Cu–Cu bumps and RDLs, the bonding strength was strong enough to sustain the thermal stress. Additionally, the middle of the Cu–Cu bumps was subjected to tension. Some triple junctions with zig-zag grain boundaries after TCT were observed. From the pre-existing tiny voids at the bonding interface, cracks might initiate and propagate along the weak bonding interface. In order to avoid such failures, a postannealing bonding process was adopted to completely eliminate the bonding interface of Cu–Cu bumps. This study delivers a deep understanding of the thermal cycling reliability of Cu–Cu hybrid joints.

## 1. Introduction

Currently, the COVID-19-pandemic-led surge in demand for high-performance computing chips is prevailing. They are used in high-end electronic devices, such as complementary metal oxide semiconductor (CMOS) image sensors [1] and high bandwidth memory (HBM) [2]. To fabricate high-performance computing chips, a three-dimensional integrated circuit (3D IC) technique is employed [3,4]. However, challenges still exist in 3D IC packaging [5], for instance, with the reliability of interconnects [6]. In a 3D IC device, solder microbumps have been widely used as interconnects between functional chips and an interposer [7]. Those solders severely suffer from electromigration [8,9,10] and thermal fatigue [11,12,13] during operations. In addition, the high resistance and downscaling of solder joints should be critically considered. Conventionally, the diameter of a solder microbump is about 30 μm, while that of a ball grid array (BGA) solder bump is about 150 μm. The volume of solder microbumps is about 125 times smaller than that of BGA solder bumps. This can lead to the severe formation of intermetallic compounds (IMCs), necking, and voids in the solder microbumps [6,14,15,16,17].

With the goal of the shrinking of joints, Cu-to-Cu direct bonding is thus adopted as a potential candidate for the next generation of interconnects. Additionally, low thermal budget bonding is the main task in 3D IC packaging. This can be achieved by low temperature or short-time bonding. To date, Cu-to-Cu direct bonding has been researched extensively [18,19,20,21]. Diffusion bonding with a (111) Cu surface [22], capping passivation layer [23], and surface activation bonding [24] were widely employed to achieve low-temperature Cu-to-Cu direct bonding.

High-strength nanotwinned Cu (nt-Cu) [25,26,27,28] was demonstrated to possess low bonding temperature and electrical resistance [28,29,30,31,32]. The (111) surface can be well controlled through electroplating [30]. The highest surface diffusivity of a (111) Cu surface can enhance the surface creep bonding during thermal compression bonding processes [22,29]. To examine the Cu-to-Cu direct bonding quality, various reliability tests were conducted [33,34]. A shear strength greater than 100 MPa was obtained by eliminating the bonding interface [33]. Thermal cycling tests (TCTs) and high-temperature storage (HTS) were conducted with a Cu/SiO_2_ hybrid bonding structure. They reported that the coefficient of thermal expansion (CTE) of SiO_2_ is smaller than that of Cu. Thus, the Cu–Cu bumps were under compression at an elevated temperature, and no obvious defects were formed [34].

To date, systematic studies on the failure mechanisms of Cu joints with high CTE dielectrics under thermal cycling are limited. In this study, the mechanical reliability and failure mechanism of samples bonded at various conditions were examined using TCT. The (111)-oriented nt-Cu was fabricated by electroplating. This research can provide helpful information to improve the current Cu/dielectric hybrid bonding technology using the high-CTE dielectrics, such as polyimide (PI) [35], polybenzoxazole (PBO) [36], benzocyclobutene (BCB) [37], and epoxy thermosets [38].

## 2. Materials and Methods

### 2.1. Sample Fabrication

The tested vehicle used in this study was designed on an 8-inch wafer, which included top-die and bottom-die patterned wafers. The Cu redistribution lines (RDLs) were first electroplated, and photosensitive PBO was used to cover the RDLs as a passivation layer by spin coating. To connect the Cu RDLs and Cu–Cu bumps, the passivation openings of PBO were fabricated through lithography. A photoresist was used to define the location and diameter of Cu–Cu bumps. An additive (Chemleader Corporation, Hsinchu, Taiwan) was employed to fabricate the nt-Cu microbumps. Prior to the Cu-to-Cu direct bonding, chemical mechanical planarization (CMP) was conducted to reduce surface roughness (*R*_q_) to 3 nm. The 8-inch wafer was then diced into various top ($6\;\times \;6\;{\mathrm{mm}}^{2}$) and bottom ($15\;\times \;15\;{\mathrm{mm}}^{2}$) dies. Various chips were fabricated by instant bonding using a chip-to-chip bonder (CA-2000VA, Bondtech Co., Ltd., Kyoto, Japan). More information about the bonding process and results can be referenced in our recent study [39].

**Figure 1:**
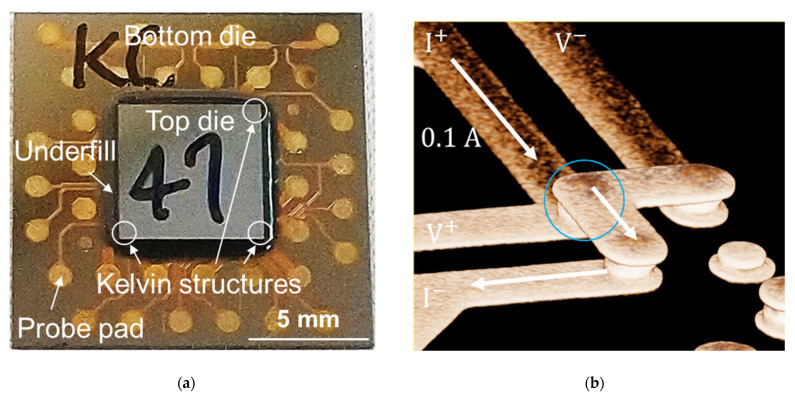
(**a**) A typical image of the as-fabricated chips. The underfill (UF) can be seen around the top die. The Kelvin structures were fabricated at the three corners of the top die. The probe pads were fabricated on the bottom die for the 4-point probe measurement. (**b**) A 3D image of the Kelvin structure.

In this study, the effects of bonding pressures and time on the electrical resistance of Cu–Cu bumps were investigated. The bonding pressure ranged from 15 to 90 MPa, with the bonding time ranging between 5 and 60 s. The bonding time was the holding time of the bonding force. After the instant bonding, the chips were dispensed with the underfill (UF) to protect the Cu–Cu bumps from oxidation during the reliability tests. Figure 1a shows a typical image of the as-fabricated chip. The UF flowed into the chips from one or two sides of the edges of the top die, and then sealed other sides of the edge. Thus, a black region (UF) around the top die can be seen in Figure 1a. The curing condition of the UF was 165 °C for 2 h.

### 2.2. Resistance Measurement of Cu–Cu Bumps

A Kelvin structure was designed and fabricated to measure the resistance of the Cu–Cu bumps at the three corners of the chip (Figure 1a). The 3D image of the Kelvin structure is shown in Figure 1b. Many probe pads (1 mm in diameter) were fabricated (Figure 1a) on the bottom die. The four-point probe technique was used to measure the electrical resistance using a power supply (Keithley 2400, Keithley Instruments, Inc., Cleveland, OH, USA). The lowest compliance level of voltage measurement was 0.2 mV. Through the probe pads, a current of 0.1 A was applied. The voltage difference of the Cu–Cu bump (blue circle in Figure 1b) could be measured. Thus, the resistance change of the single Cu–Cu bumps could be observed during TCT. Four chips were fabricated for each bonding condition to conduct TCT, with a resistance change among the Cu–Cu bumps averaging 12 Cu–Cu bumps. Thus, we can avoid processing variation and measurement error.

### 2.3. Thermal Cycling Test

**Figure 2:**
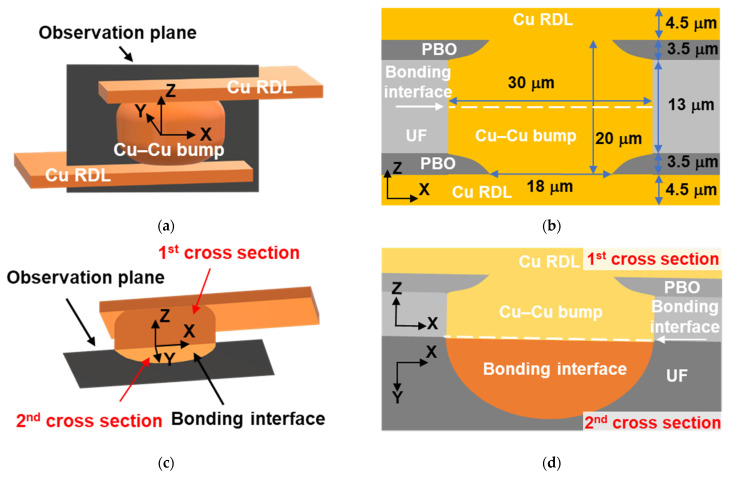
Illustrations of two kinds of FIB cross-sectional images. (**a**,**c**) Three-dimensional schematics of the Cu–Cu bumps and RDLs. The coordinates were marked at the middle of the Cu–Cu bumps. The observation plane in (**a**) was used to show the cross-sectional images (**b**). (**b**) is the first cross section of the *x*-*z* plane and shows the dimensions of the Cu–Cu bump and RDLs, which were used in FEM. The observation plane in (**c**) was employed to present the cross-sectional images (**d**). (**d**) The cross sections contain the *x-z* and *x-y* planes, showing the bonding interface in half spheres. The *x-y* plane is the second cross section.

In order to study the TCT reliability of the chips, four different bonding conditions were chosen, as listed in Table 1. After the bonding process, those samples underwent TCT (TCC-150W, ESPEC Co., Osaka, Japan). The TCT temperature ranged from −55 to 125 °C. The soak time was 5 min, and the ramp rate was 18 °C/min. The TCTs were terminated after 1000 cycles. The resistance of the Cu–Cu bumps was measured after each set of 250 thermal cycles. The criterion of resistance change was 20%. Two types of cross-sectional focused ion beam (FIB) images (Figure 2) were employed to characterize the failure mechanisms. After TCT, the chips were grinded and polished at the site of Kelvin bumps to study the TCT failure mechanisms. The FIB was used to ion-mill the first cross section (Figure 2a,b). Those chips were then vertically attached on the FIB holder for the second ion milling of the second cross section (Figure 2c,d). Additionally, the electron images were used to detect voids and cracks, while the ion images were employed for Cu grain analysis. The finite element method (FEM, ANSYS Workbench, Canonsburg, Pennsylvania, USA) was utilized to construct the models with the single Cu–Cu bumps and analyze their stress distribution. The detailed parameters of the FEM models of single Cu–Cu bumps with Cu RDLs and a Si substrate are shown in Figure 2b and in reference [40]. In order to simulate the stress in the corner Cu–Cu bumps of the chips, two sides of the model were set as symmetry regions. The stress distributions at −55 and 125 °C were then discussed. Additionally, a scanning transmission electron microscope (STEM) was used to characterize the void distribution of the bonding interface. Using the FIB, STEM images, and numerical analyses, the relationships between cracking, void formation, Cu grains, and stresses were then correlated.

## 3. Results and Discussion

### 3.1. As-Fabricated Samples with Different Bonding Conditions

#### 3.1.1. Electrical Resistance Measurement

**Figure 3:**
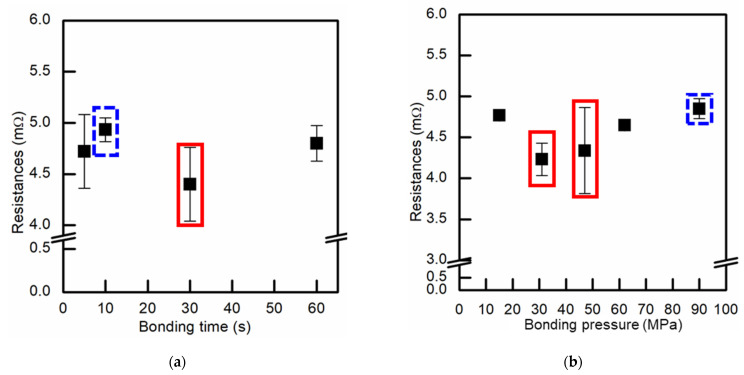
Resistances of the Cu–Cu bumps bonded at different bonding conditions. (**a**) The bonding temperature and pressure were kept at 300 °C and 90 MPa, while the bonding time ranged from 5 to 60 s. (**b**) The bonding temperature and time were kept at 300 °C and 10 s, while the bonding pressure ranged from 15 to 90 MPa.

The resistances of the Cu–Cu bumps bonded at different conditions are shown in Figure 3. The resistance was measured after the bonding process without UF dispensing. With the same bonding temperature (300 °C) and pressure (90 MPa), the bonding time could be shortened from 60 to 5 s (Figure 3a). If the bonding temperature and time were kept at 300 °C and 10 s, respectively, the bonding pressure could be decreased from 90 to 15 MPa (Figure 3b). The bonding condition (the dash blue square) was 300 °C/90 MPa/10 s. As the bonding temperature was 300 °C, the resistance of the single Cu–Cu bump could remain around 4.5 mΩ, even though the bonding pressure or time was lower. Therefore, the two higher bonding pressure (300 °C/90 MPa/30 and 10 s) and two lower bonding pressure (300 °C/47 and 31 MPa/10 s) conditions were chosen to carry out TCTs. Those four conditions are marked with the blue dashed line and red solid line squares in Figure 3 and listed in Table 1.

#### 3.1.2. Microstructure of Cu–Cu Bumps

**Figure 4:**
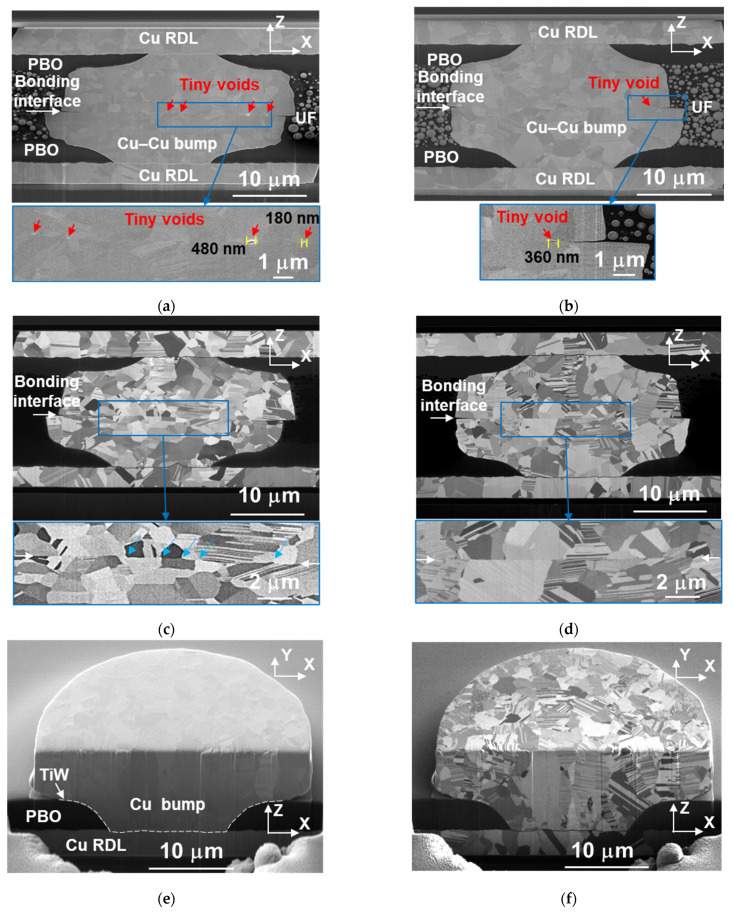
Cross-sectional images of the as-fabricated Cu–Cu bumps bonded at (**a**,**c**) 300 °C/90 MPa/30 s and (**b**,**d**) 300 °C/31 MPa/10 s. (**a**,**b**) and (**c**,**d**) are electron and ion images, respectively. (**a**–**d**) The bonding interface in the blue squares was enlarged. (**a**,**b**) The lenticular shape of voids can be observed. (**c**,**d**) The grain boundary at the bonding interface can be observed. White arrows point out the bonding interface, and blue arrows point out the triple junctions. (**e**,**f**) Three-dimensional images of the Cu bumps after CMP and before the bonding process. (**e**,**f**) are electron and ion images, respectively.

The cross-sectional FIB images of the as-fabricated Cu–Cu bumps bonded at 300 °C/90 MPa/30 s and 300 °C/31 MPa/10 s are shown in Figure 4a,c and Figure 4b,d, respectively. The electron images in Figure 4a,b show that the Cu RDLs connected with the Cu–Cu bumps, and the PBO covered the RDLs acting as passivation layers. The Cu–Cu bumps were surrounded by UF for excellent filling. Some white particles in UF are fillers, which were mixed into epoxy thermosets to obtain better mechanical properties. At the bonding interfaces of the Cu–Cu bumps, some tiny voids existed after the instant bonding process. These void formations might be attributed to the defects on the Cu surfaces. They transformed into various lenticular shapes (Figure 4a,b) due to the pressure gradient and Gibbs–Thomson effect during thermal compression bonding process [41]. The length of the voids was measured, with the largest value being 480 nm. Other voids were too small to be measured through SEM images, and this will be discussed in Section 3.5.

The 3D images of the Cu microbumps after CMP are shown in Figure 4e,f. A TiW adhesion layer between the PBO, Cu RDL, and Cu microbump is shown in Figure 4e. The nt-Cu columnar grains can be observed at the middle and edge of the Cu microbumps [39]. Compared with the distribution of grains in Figure 4c,f, because of the high bonding pressures (90 MPa) and temperature (300 °C), the nt-Cu columnar grains recrystallized to form fine grains during the bonding process. Therefore, more recrystallized grains can be observed in Figure 4c than in Figure 4d. However, the bonding time (30 and 10 s) was not long enough for grain growth. We found that more nt-Cu columnar grains remained in Figure 4d compared with those in Figure 4c due to the lower bonding pressure and time.

The distribution of grains is also presented in the enlarged images of the bonding interfaces (blue squares in Figure 4c,d). Some triple junctions [42,43] were detected (blue arrows in Figure 4c). These triple junctions led to a zig-zag bonding interface. This indicates that the atomic interdiffusion at the bonding interface occurred in the sample bonded at 300 °C/90 MPa/30 s. This phenomenon might be caused by the recrystallization of grains at the bonding interface (Figure 4c). These slightly grew further across the bonding interface. However, the bonding interface bonded at 300 °C/31 MPa/10 s still exists as a straight line (Figure 4d). If the bonding time is not extended to minutes or hours, the bonding interface will not be eliminated [18,33].

### 3.2. Resistance Change of the Cu–Cu Bumps under Thermal Cycling

**Figure 5:**
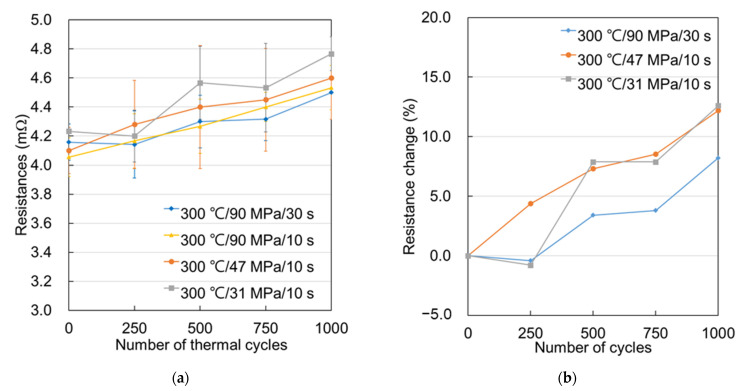
The resistances of the Kelvin bumps were measured after each set of 250 thermal cycles. Average (**a**) resistance and (**b**) resistance change for each bonding condition.

The resistance change of the Cu–Cu bumps under TCT is shown in Figure 5. Note that the as-fabricated Cu–Cu bumps bonded at different bonding conditions underwent UF dispensing and full curing. Their initial electrical resistances were denoted as 0 cycle (Figure 5a), and the values are from 4.0 to 4.4 mΩ, which were set as the 0% resistance change for each bonding condition. After 250 cycles, the resistance of the Cu–Cu bumps bonded at 300 °C/47 MPa/10 s increased by 4.4%, and those bonded at 300 °C/90 MPa/10 s increased by 2.7%, while the others did not change. The resistance significantly increased after 500 cycles and reached 8–12% after 1000 cycles. Using a 20% criterion, all of the bonding thus passed 1000 thermal cycles. At first glance, the scale of TCT damage of the three bonding conditions appeared with only a 4% difference of resistance change after 1000 thermal cycles. However, the actual damage was totally different, and is confirmed in the following cross-sectional images.

### 3.3. Cross-Sectional Images of TCT Damage

**Figure 6:**
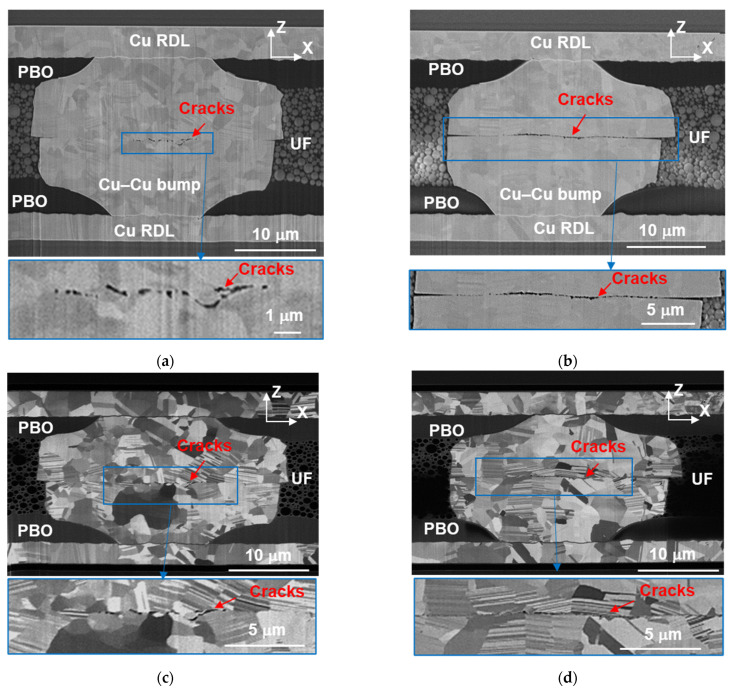
Cross-sectional images of the as-fabricated Cu–Cu bumps bonded at (**a**,**c**) 300 °C/90 MPa/30 s and (**b**,**d**) 300 °C/31 MPa/10 s. (**a**,**b**) and (**c**,**d**) are electron and ion images, respectively.

**Figure 7:**
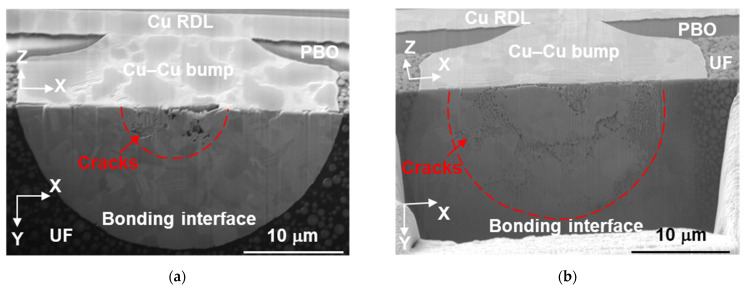

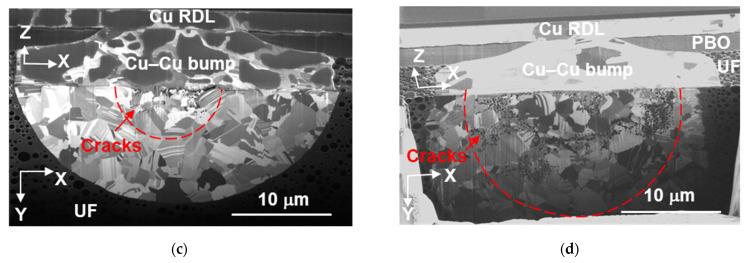
The second cross-sectional images of the as-fabricated Cu–Cu bumps bonded at (**a**,**c**) 300 °C/90 MPa/30 s and (**b**,**d**) 300 °C/31 MPa/10 s. (**a**,**b**) and (**c**,**d**) are electron and ion images, respectively.

Two kinds of the cross-sectional images of the Cu–Cu bumps after 1000 cycles are shown in Figure 6 and Figure 7.

#### 3.3.1. The First Cross-Sectional Images

Figure 6a,b shows the electron images in the *x-z* plane. Various cracks are present at the bonding interface. There is no gap among the UF, PBO, RDLs, and Cu–Cu bumps. The Cu–Cu bumps are uniformly surrounded by the UF. The cracks at the bonding interface in Figure 6a are much smaller than those in Figure 6b, but their difference in resistance change is only 4%. Some Cu grains recrystallized, and further little grains grew at the center of the bonding interface. Several zig-zag cracks propagated along the fine grains in Figure 6c. In contrast, the cracks are straighter in Figure 6d because the grains did not recrystallize and grow across the weak grain boundaries to form triple junctions at the bonding interface [42,43]. This difference was caused by the microstructure of as-fabricated Cu–Cu bumps in Figure 4c,d. In Figure 4c, there are many recrystallized fine grains at the bonding interface after the 300 °C/90 MPa/30 s bonding with some remaining triple junctions. During TCTs, those fine grains grew further across the bonding interface. On the contrary, the as-fabricated Cu–Cu bumps bonded at 300 °C/31 MPa/10 s did not have many recrystallized fine grains at the bonding interface. Thus, their bonding interfaces were straight and weak, which led to severe cracking after 1000 thermal cycles.

#### 3.3.2. The Second Cross-Sectional Images

To obtain a more precise cracking region at the bonding interface, the same Cu–Cu bumps were further FIB-cut, and the second cross sections in the *x-y* plane are shown in Figure 7. The observation plane is shown in Figure 2c, and the correlation of the first and second cross sections is shown in Figure 2d. We found that cracks confined in a circle located at the bonding interface in Figure 7a,b. The cracking profile appears as a ductile fracture surface. The interfacial cracks seem to be independent of grain boundaries in Figure 7c,d; however, various zig-zag cracks initiated and propagated along grain boundaries in Figure 6c,d). Some dark stains on the *x-z* plane (Figure 7a,c) are Cu oxides. These appeared after a few days of sample storage between the first and second cross section analyses.

The cracking area in Figure 7a is smaller than that in Figure 7b, corresponding to a 4% difference in resistance change. It has been reported that the resistance increased by 5% as the contact ratio increased from 20% to 100% [44]. In this study, the resistance change is approximately 8–12% after 1000 cycles, and the crack distribution is very different (Figure 7a,b). The scale of the cracking area depends on the bonding pressure and bonding time. Cracks are located in a 5.4 μm radius circle in Figure 7a as bonded at 300 °C/90 MPa/30 s. For the Cu microbump bonded at 300 °C/31 MPa/10 s, it is 11 μm and is shown in Figure 7b. The remaining bonding ratio accounts for 87% and 46% as bonded at 300 °C/90 MPa/30 s and 300 °C/31 MPa/10 s, respectively. Therefore, the damage mechanisms are strongly related to the bonding conditions and are not easily correlated to the difference in resistance change.

### 3.4. Finite Element Method

**Figure 8:**
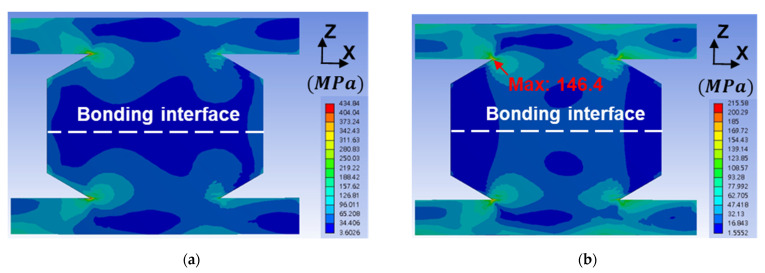
Equivalent stress (von Mises) distribution in the FEM Cu–Cu bumps and RDLs at (**a**) −55 °C and (**b**) 125 °C.

**Figure 9:**
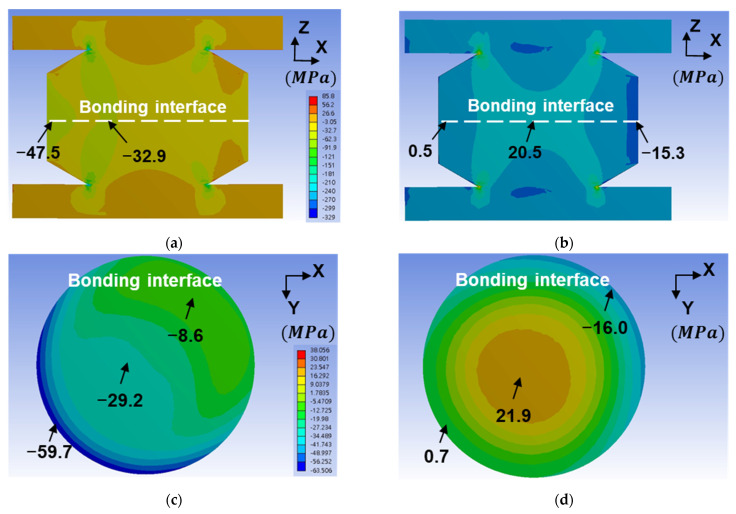
Stress distribution in the FEM Cu–Cu bumps and RDLs at (**a**,**c**) −55 °C and (**b**,**d**) 125 °C. (**a**,**b**) and (**c**,**d**) are stress distributions in the *x-z* and *x-y* planes, respectively.

Normally, cracks initiate at the edge and propagate along grain boundaries or an interface [11,12,13]. Interestingly, we found that the cracks formed along the grain boundaries at the bonding interface center. Thus, we constructed the FEM models to elucidate such a phenomenon. The parameters of material properties for FEM are listed in Table 2. The equivalent stress, or von Mises stress, distribution in the Cu–Cu bumps and RDLs is shown in Figure 8. The applied temperatures in Figure 8a,b are −55 and 125 °C, respectively. The location of maximum stress at 125 °C is at the corner of the Cu–Cu bump and RDL, and the stress is 146.6 MPa. However, there is no crack or void at that site in Figure 6, with the actual sites of the cracks in Figure 6 and Figure 7 showing at the bonding interface. The stress distributions normal to the bonding interface, which is along the *z*-axis, are shown in Figure 9.

At −55 °C, the bonding interface is under compression with a maximum stress of 59.7 MPa at the edge, which is shown in Figure 9a,c. At 125 °C, the interface is under tension with a maximum stress of 21.9 MPa, which is shown in Figure 9b,d. Interestingly, the edge of the bonding interface is under compression. Obviously, the formation of interfacial cracks is attributed to the tensile stresses shown in Figure 9b,d. In Table 2, the CTEs of the dielectrics (PBO and UF) are four and two times greater than that of the Cu. At 125 °C, the middle of the Cu–Cu bumps is subjected to tensile stresses. Note that the tensile yield stress of the annealed Cu is ~100 MPa [28]. Thus, the plastic deformation at the bonding interface will not occur at 125 °C. However, thermal fatigue can lead to crack formation initiating from the pre-existing defects at the bonding interface. In addition, the bonding interface is considered the weakest boundary of the instant bonding. Thus, cracks initiate and propagate at the middle region. If the bonding strength is strong enough, the cracking region caused by thermal cycling is smaller at the central bonding interface, as shown in Figure 6a,c; Figure 7a,c; and Figure 9b,d.

### 3.5. Defects at Bonding Interface for Crack Formation

According to the SEM images and FEM analyses, the maximum stress (Figure 8b) was located near the RDLs, but the cracks formed at the bonding interface (Figure 6 and Figure 7). It is reasonable that there was a TiW adhesion layer between Cu–Cu bumps and RDLs. Its adhesive strength was strong enough to sustain the thermal stress. On the other hand, many triple junctions were observed in Figure 4c, but cracks still formed at the bonding interface shown in Figure 6a. The reason is that some defects at the bonding interface were not detected by the SEM images.

**Figure 10:**
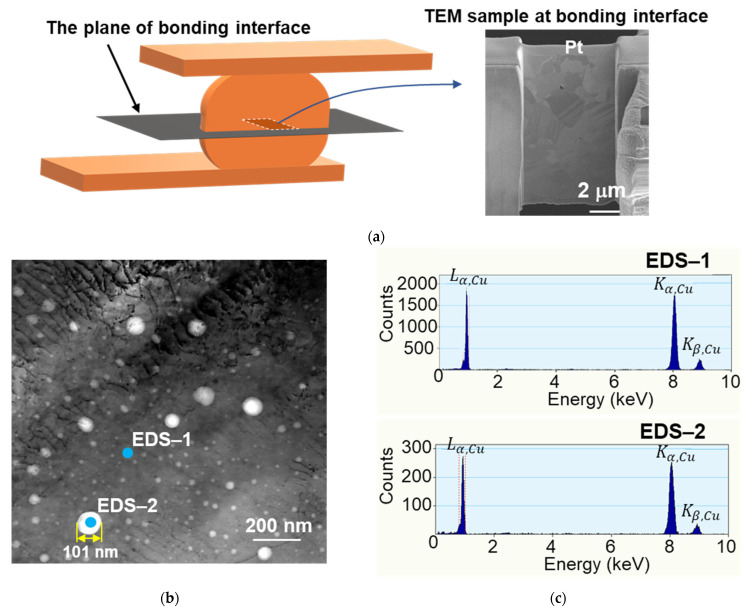
Typical STEM images of the as-fabricated Cu–Cu bumps bonded at 300 °C/90 MPa/30 s showing the bonding interface. (**a**) Schematic of the cutting site of the TEM sample. The thickness of the sample is 100–150 nm containing various defects on the bonding interface. (**b**) Typical bright field STEM image of the bonding interface. (**c**) EDS spectrums taken at site-1 and site-2 in (**a**). The energy of site-1 is much stronger than that of site-2; thus the white dot at site-2 can be considered a void.

In our previous study [42], the cross-sectional TEM images show that some voids were located at the bonding interface. Such voids are the weak points for crack initiation during TCT. Cracks might initiate from those voids and propagate along the bonding interface. In this study, the STEM image was used to investigate the void distribution. In order to focus on the bonding interface, a TEM sample containing a bonding interface was cut from the Cu–Cu bumps bonded at 300 °C/90 MPa/30 s (Figure 10a). The thickness of the TEM sample is ~100−150 nm, so the voids at the bonding interface could be easily observed. The bright field STEM image (Figure 10b) shows many white dots segregated at the bonding interface. The largest diameter of these white dots is ~100 nm. Energy dispersive X-ray spectroscopy (EDS) spectrums are shown in Figure 10c. With the same analysis time, the counts of the signal strength of $K_{\alpha ,\;Cu}$ and $L_{\alpha ,\;Cu}$ are between 1500 and 2000 at the site of the EDS-1, while those at the EDS-2 range from 200 to 300. The white dot can be certified as a void. Due to the segregated distribution of voids and the tensile stress at the center of the bonding interface (Figure 9b,d), cracks are likely to form at the bonding interface.

In this study, the dielectric around the Cu–Cu bumps is mainly composed of UF. It can be replaced by PI, BCB, or other polymer dielectrics [45,46,47,48]. The CTE of these dielectrics is larger than that of Cu. When the chip temperature is higher than its glass transition temperature (*T*g), the CTE might be larger than 100 ppm/°C; however, Young’s modulus will decrease. If the Cu–Cu bumps are under tension, cracks will form at the weak bonding interface. To suppress such a crack formation, longer bonding is needed for bonding interface elimination. The dielectrics have a potential for hybrid bonding using instant bonding [39] and postannealing [49] to enhance its reliability.

## 4. Summary

In summary, we studied the failure mechanisms of the Cu–Cu bumps with underfill during TCT. The resistance change of the Cu–Cu bumps was 8–12%, which was lower than the 20% criterion. Due to the high CTE of the dielectrics and weak grain boundaries of the bonding interface, cracks formed at the middle of the Cu–Cu bumps. The crack scale decreases with the increase in bonding pressure and time.

We found that some triple junctions formed at the bonding interface of the bump bonded at 300 °C/90 MPa/30 s. These triple junctions implied that the Cu atoms diffused across the bonding interface and became a zig-zag grain boundary after TCT. Many voids at the bonding interface were observed using STEM. The FEM results show that, at 125 °C, the maximum stress locates at the Cu RDLs. Due to good adhesion between the TiW layer with the Cu bump and RDLs, cracks rather formed at the bonding interface. Under thermal cycling, cracks might initiate from the pre-existing voids and propagate along the bonding interface.

When bonded at 300 °C/31 MPa/10 s, cracks in the bumps caused by thermal cycling were more serious than those of higher bonding pressure and longer time. The bonding interface was quite straight, indicating less Cu atomic diffusion across the bonding interface. A two-step bonding, which includes instant bonding and postannealing, is suggested to strengthen the bonding interface for greater mechanical reliability by eliminating the weak bonding interface.

## Figures and Tables

**Table 1:** Four bonding conditions chosen for the TCTs.

No.	Atmosphere	Temperature (°C)	Bonding Pressure (MPa)	Bonding Time (s)
1	N_2_ ambient	300	90	30
2	N_2_ ambient	300	90	10
3	N_2_ ambient	300	47	10
4	N_2_ ambient	300	31	10

**Table 2:** Material properties used in the FEM models.

Material	Poisson’s Ratio	Thermal Conductivity (W/m°C)	*T*g (°C)	CTE (ppm/°C)	Young’s Modulus (GPa)
Cu	0.34	401	-	16.8	110
PBO	0.3	0.2	300	64	2.3
Underfill	0.3	0.4	130	30 (<Tg) 110 (>Tg)	0.2 (<Tg) 8.5 (>Tg)
